# Cytocompatible and multifunctional polymeric nanoparticles for transportation of bioactive molecules into and within cells

**DOI:** 10.1080/14686996.2016.1190257

**Published:** 2016-07-06

**Authors:** Kazuhiko Ishihara, Weixin Chen, Yihua Liu, Yuriko Tsukamoto, Yuuki Inoue

**Affiliations:** ^a^ Department of Materials Engineering, School of Engineering, The University of Tokyo, Tokyo, Japan; ^b^ Department of Bioengineering, School of Engineering, The University of Tokyo, Tokyo, Japan

**Keywords:** Nanomedicine molecular science, zwitterionic polymer, fluorescence property, cytocompatibility, surface modification, in-cell device, 30 Bio-inspired and biomedical materials, 600 Biomaterials, Nano materials

## Abstract

Multifunctional polymeric nanoparticles are materials with great potential for a wide range of biomedical applications. For progression in this area of research, unfavorable interactions of these nanoparticles with proteins and cells must be avoided in biological environments, for example, through treatment of the nanoparticle surfaces. Construction of an artificial cell membrane structure based on polymers bearing the zwitterionic phosphorylcholine group can prevent biological reactions at the surface effectively. In addition, certain bioactive molecules can be immobilized on the surface of the polymer to generate enough affinity to capture target biomolecules. Furthermore, entrapment of inorganic nanoparticles inside polymeric matrices enhances the nanoparticle functionality significantly. This review summarizes the preparation and characterization of cytocompatible and multifunctional polymeric nanoparticles; it analyzes the efficiency of their fluorescence function, the nature of the artificial cell membrane structure, and their performance as in-cell devices; and finally, it evaluates both their chemical reactivity and effects in cells.

## Graphical abstract image

Cells can uptake polymer nanoparticles, embedding quantum dots covered with cytocompatible phospholipid polymers and cell-penetrating peptides.

**Figure:**
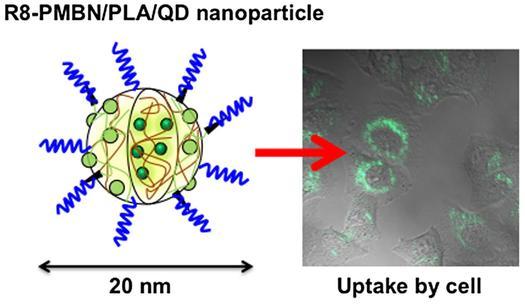

## 1. Introduction

Nanoparticles have remarkable potential and find broad applications industrially as well as academically due to their various functionalities.[1–6] They are obtained from metals, metal oxides, metallic alloy, and polymers and they show unique physical and chemical characteristics compared with those of bulk materials. Nanoparticles can be dispersed in solvents to provide stable suspensions due to interactions between the nanoparticle surfaces and the surrounding solvent molecules. The large surface-to-volume ratio of nanoparticles induces high efficiency when applied as catalysts. Depending on the size of the nanoparticles, certain metal nanoparticles will display surface plasmon resonance and magnetic nanoparticles will display superparamagnetism. Moreover, assemblies of nanoparticles provide unique structures with various functions.[7–13] The nanometer scale of these structures makes them suitable for analysis of microscopic areas, such as targeted intracellular regions. Nanoparticles are actively used in pharmaceutical and biomedical applications as carriers for biomolecules to facilitate efficient delivery into cells and tissues.

However, the use of nanoparticles in biological systems is often limited by their specific structures. Nanoparticles possess high-energy surfaces and can therefore lead to aggregation of colloidal particles by attractive van der Waals forces or electrostatic interactions. Random adsorption of proteins on nanoparticle surfaces reduces the functionality according to the size effect. In a cell culture medium, nanoparticles are adsorbed onto the cell membrane and are internalized by the cell through endocytosis. Under biological conditions, regardless of the nature of the application, nanoparticles occasionally induce unexpected cellular responses with disrupted functionalities because of nonspecific protein adsorption and the corresponding biological responses. To overcome these problems, nanoparticle surfaces are engineered to render them more biocompatible and bioinert.[14–19]

Several methodologies are available to obtain bioinert surfaces on nanoparticles, including surface modification with natural macromolecules [20–23] or water-soluble synthetic polymers. Water-soluble synthetic polymers, such as poly(ethylene glycol) (PEG) and its derivatives, are used for grafting or coating the nanoparticle surface.[24,25] These polymer chains expand into the aqueous medium, generating a highly mobile hydrated layer that surrounds the nanoparticles. The steric hindrance generated by the polymer chains disturbs aggregation of the nanoparticles and prevents undesirable biological reactions. Recently, however, some articles have reported accelerated blood clearance of PEG-modified nanocarriers, and immunoresponses such as production of immunoglobulin M by PEG. Thus, it is necessary to note this point when PEG is used as a surface modification reagent for nanoparticles.[26–28]

**Figure 1.**
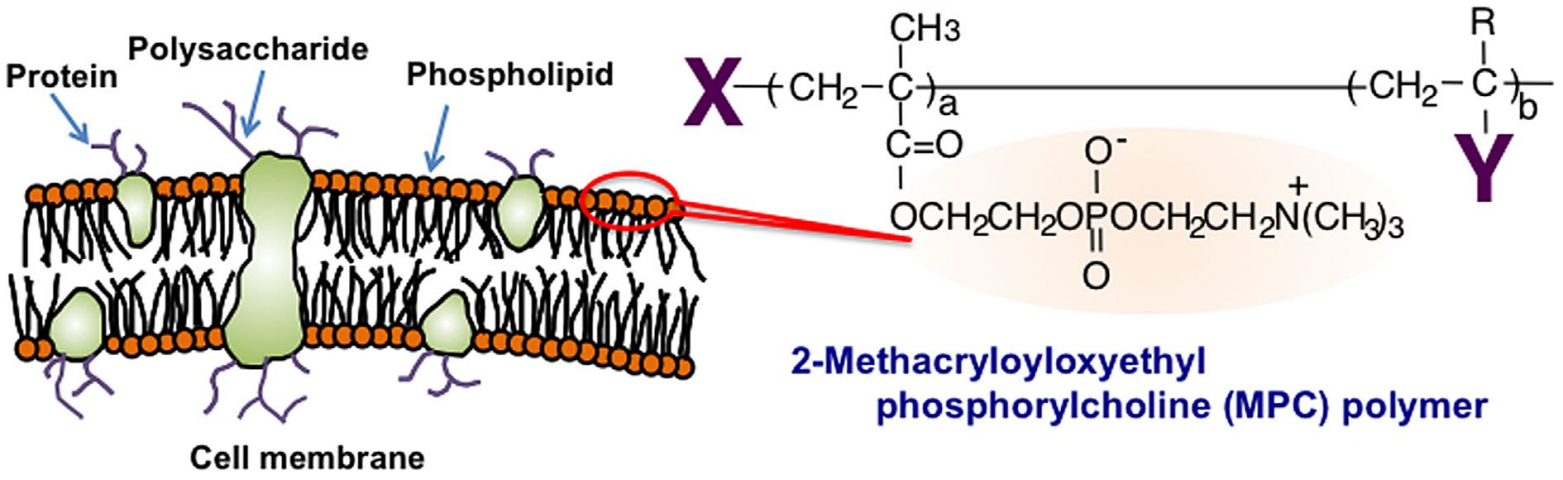
Cell membrane-inspired molecular design and chemical structure of MPC polymer.

Other methodologies have been investigated for surface modifications using phospholipid derivatives such as phosphatidylcholine derivatives. Although they are used as pharmaceutical carriers of bioactive reagents in the bloodstream, their stability is insufficient to enable long-term circulation in the bloodstream. Hence, a polymerization technique is a promising method to improve the stability of the phospholipid assembly. Phospholipid derivatives with polymerizable groups have been synthesized.[29–32] One of the most effective of these is 2-methacryloyloxyethyl phosphorylcholine (MPC), which possesses both a methacrylate group and a monomer, and accordingly, various kinds of MPC polymers have been used to synthesize the zwitterionic phosphorylcholine group.[33–35] MPC is polymerized easily by radical polymerization, even in the presence of other monomers (Figure 1). The MPC polymers are hydrophilic and are electrically neutral due to their unique zwitterionic structure. They have good stability under biological circumstances, namely, they are biologically inert at pH 7.4 and under high ionic strength. They have excellent biocompatibility, specifically resistance against protein adsorption, cell adhesion, and tissue immunoreactions.[36–42] Thus, based on these fundamental characteristics, selected MPC polymers are used as surface modification materials in medical devices, such as implantable artificial hearts and artificial hip joints.[43–48]

Considering the functional properties of MPC polymers and their usefulness in providing a bioinert platform on the nanoparticle surface, the present review focuses on elucidating precisely designed MPC polymers and adding insight into the recent promising results related to the fabrication of nanoparticles for in-cell imaging.

## 2. Surface modification of nanoparticles with MPC polymers

Substantial research data exists related to modification of nanoparticle surfaces by MPC polymers. Table 1 summarizes the various nanoparticle systems using MPC polymers. In such preparations, the components used as core materials include biologically active compounds, inorganic nanoparticles, and polymer nanoparticles. The nanoparticle diameters range from a few nanometers to a few hundred nanometers. The amphiphilic nature of MPC polymers induces formation of polymer aggregates in an aqueous medium, along with the generation of a hydrophobic region, thereby facilitating entrapment of hydrophobic bioactive compounds including anti-cancer compounds.[81,82] MPC polymer aggregates are stable when injected into the bloodstream and concentrate in cancer tissue, triggering a dramatic anti-cancer effect. This delivery system is ideal for transporting poorly water-soluble anti-cancer compounds into living systems.[49–52] Additionally, inorganic nanoparticles can largely be dispersed in an aqueous medium by surface modification with water-soluble MPC polymers. Contemporary progress in the field of polymer synthesis has established the surface-initiated living radical polymerization procedure, which could be applied to MPC associated surface modifications of nanoparticles.[83,84] During the polymerization process, the living radical initiator is immobilized onto the surface of inorganic nanoparticles and living radical polymerization is carried out to generate a poly(MPC) grafted layer.[60,63,66,70,73] The poly(MPC)-grafted chains have nearly the same degree of polymerization and hence the same chain length. This indicates that the diameter distribution of the poly(MPC)-grafted nanoparticles depends on the original core nanoparticles, and therefore uniform functionalities are expected. In addition, modified nanoparticles could efficiently disperse in an aqueous medium.

**Table 1.** List of nanoparticles covered with MPC polymers

**Core material**	**MPC polymer**	**Diameter (nm)**	**Functions**	**References**
**Bioactive molecules**				
Pacritaxel	PMBN-EGF	50–75	Anti-tumor effect	49
Pacritaxel	PMBN-per S1	50	Anti-tumor effect	50
Doxorubicin	P(MPC-*co*-EtOEMA)	—	Anti-tumor effect	51
Griseofulvin	PMB30	20–50	Pharmaceutical activity	52
Tranilast	PMB30	*c*.100	Solid dispersion	53
Cyclosporine A	PMB30	*c*.180	Solid dispersion	54
Plasmid DNA	PMB50	190 ± 3	Transfection effect	55
DNA/β-CD complex	PMPC-adamantyl	110–150	Gene carrier	56
DNA	PMD-folic acid	100–165	Gene carrier	57
CHP	PMPC	59.7 ± 27.4	Self-aggregation	58
**Inorganic nanoparticles**				
CaCO_3,_ BaCO_3_	PMPC	1,000–3000	Biomineralization	59
Graphene oxide	PMPC	500–1500	Biomedical application	60
Magnetic particle	PMPC	*c*.400	Immuno-reaction	61
Fe_3_O_4_ nanoparticle	PMPC	18.7 ± 1.5	Contrast agent of MRI	62
Fe_3_O_4_ nanoparticle	PMPC	8.9 ± 0.1	Contrast agent of MRI	63
Fe_3_O_4_/SiO_2_	P(MPC-*co*-AAm)	630–670	Molecular imprinting	64
SiO_2_	PMPC	12	Stabilizer	65
Mesoporous SiO_2_	PMPC	<1100	In cell carrier	66
TiO_2_ nanoparticle	PMPC	100	Suspension in aqueous media	67
Ag nanoparticle	P(MPC-*co*-HEMA)	130–160	Thin film formation	68
Ag nanoparticle	P(MPC-*co*-HEMA)	100–500 (Film)	Anti-bacterial activity	69
Au nanoparticles	PMPC	100–300	Protein detection	70
Au nanorod	P(MPC-*co*-DHLA)	17.8 ± 1.8 × 53.1 ± 3.5	Imaging in cell	71
Pd nanoparticle	PMPC-*block*-PDiPAMA	*c*.20–30	Catalyst for coupling	72
CdSe/ZnS QD	PMPC	12	Imaging in cell	73
CdSe/ZnS QD	PDbNbM	20–30	Tracking in cell	74
**Polymer nanoparticles**				
Polystyrene	PMPC-NH_2_	333–611	Template for Au nanoparticle	75
Poly(lactic acid)	PMBN	250–300	Immobilization of IgG	76
Poly(lactic acid)	PMB30	400–500	Cell-based assay	77
Poly(lactic acid)	PMB-PL	127	Photoinduced release of protein	78
Poly(amido amine)	PMPC	115–156	Dendorimer dispersion	79
Poly(BMA)	PMPC	100	Near-IR imaging	80

PMPC: poly(MPC), PMB50: water-soluble poly(MPC-*co*-BMA) MPC unit content 50 mol%, PMBN: poly(MPC-*co*-BMA-*co*-MEONP), CHP: cholesteryl groups-bearing pullulan, P(MPC-*co*-HEMA): poly(MPC-*co*-hydroxyethyl methacrylate), P(MPC-*co*-DHLA): poly(MPC-*co*-methacryloyloxyethyl dihydrolipoic acid), P(MPC-*co*-AAm): poly(MPC-*co*-acrylamide), P(MPC-*co*-EtOEMA): poly(MPC-*co*-2-ethoxy-2-oxyethyl methacrylate), PMB30: water-soluble poly(MPC-*co*-BMA) MPC unit content 30 mol%, molecular weight 5.0 × 10^4^, PMD: poly(MPC-*co*-N,N-dimethylaminoethyl methacrylate (DMAEMA)), PDbNbM: poly(DMAEMA)-*block*-poly(MEONP)-*block*-poly(MPC), PMB-PL: poly(MPC-*co*- BMA-*co*-4-(4-(1-methacryloyloxyethyl)-2-methoxy-5-nitrophenoxy)butyric acid), PMPC-*block*-PDiPAMA: poly(MPC-*block*-2-(N,N-diisopropylamino)ethyl methacrylate).

## 3. Surface modification of quantum dots with MPC polymers

Semiconductor nanoparticles such as quantum dots (QDs) have received strong interest as a promising material in biological imaging research, and can serve as alternatives to organic fluorescent dyes.[85–89] QDs are typically between 2 and 6 nm and have unique optical properties depending on the size, such as fluorescent multi-color emission spectra, high quantum yields, and excellent resistance to photobleaching by excitation light. These optical properties are suitable in most fluorescence applications, particularly for long-term monitoring of labeled substances, an area in which QDs have a singular advantage over conventional fluorescent organic dyes.

*In vitro* biological applications of QDs include cell labeling and tracking cell migration, and *in vivo* they are applied as contrast agents in tumor-tissue sections.[90–93] The QD surface is generally covered by trioctylphosphine oxide (TOPO) and *n*-octylamine derivatives to form a stable hydrophobic layer.[94] QDs are difficult to disperse in aqueous medium themselves and sometimes they induce serious damage to the target cells and tissues. Thus, the surfaces of QDs must be modified with hydrophilic or water-soluble compounds to achieve stable and highly sensitive bioimaging without cytotoxicity in cell culture medium.

**Figure 2.**
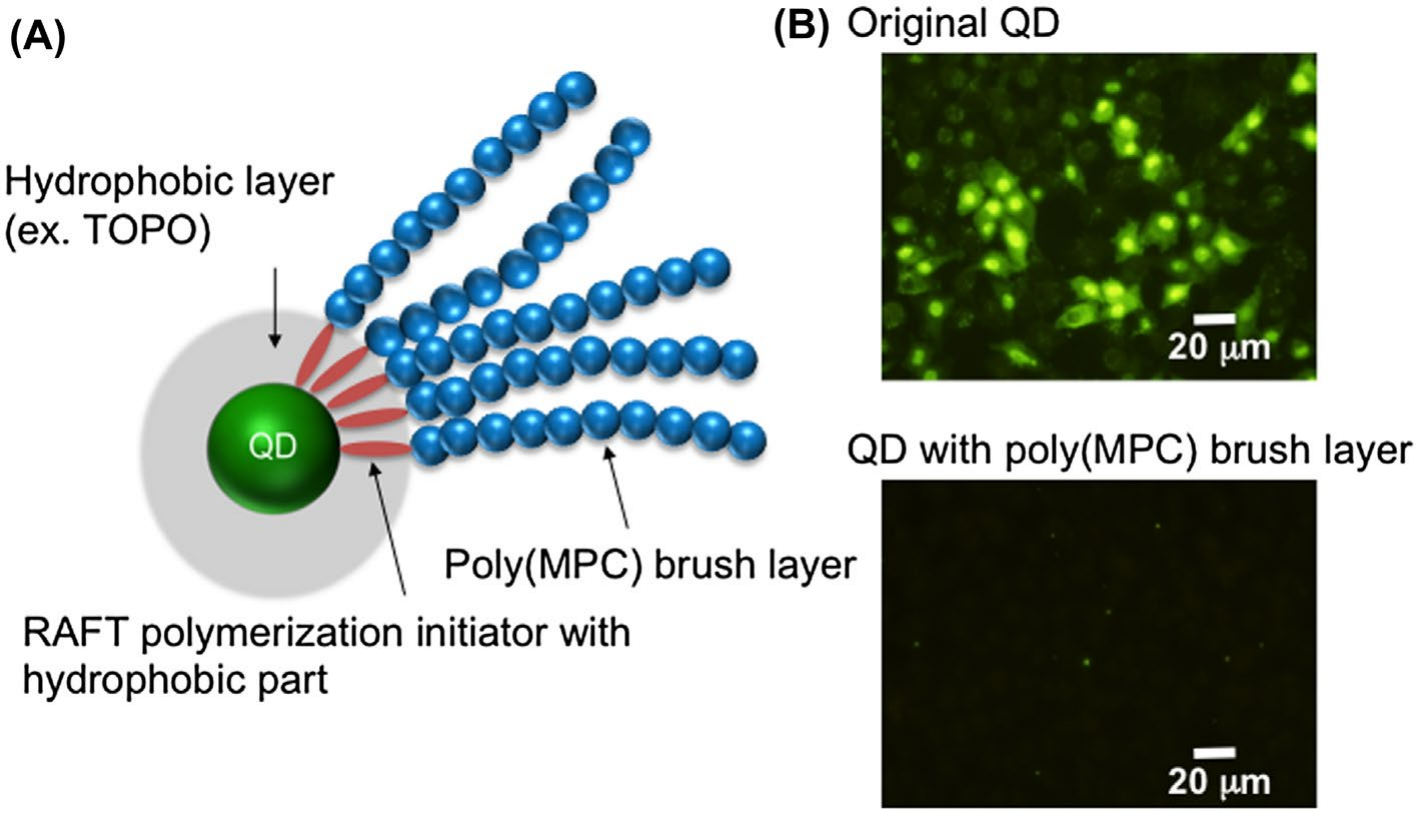
Schematic representation of QD modified with poly(MPC) (A) and fluorescence microscopy image of QD internalization into cells (B).

Preparation of QDs modified with the MPC polymer was reported initially by Matsuno and coworkers [73]. They successfully grafted poly(MPC) chains onto QD (CdSe/ZnS) surfaces using reversible addition-fragmentation chain transfer (RAFT) polymerization (Figure 2). In this process, an amphiphilic RAFT polymerization initiator forms a micelle-like structure in an aqueous solution and solubilizes the QDs that are covered with a TOPO layer. Subsequently, at the surfaces of QDs treated with the RAFT polymerization initiator, surface-initiated RAFT polymerization of MPC can occur under normal RAFT polymerization conditions. The poly(MPC) chains formed are then immobilized stably on the surface. The QDs modified with poly(MPC) chains show good cytocompatibility and are able to inhibit uptake by HeLa cells, although the QDs modified with poly(MPC) chains have very small diameters (~12 nm).

**Figure 3.**
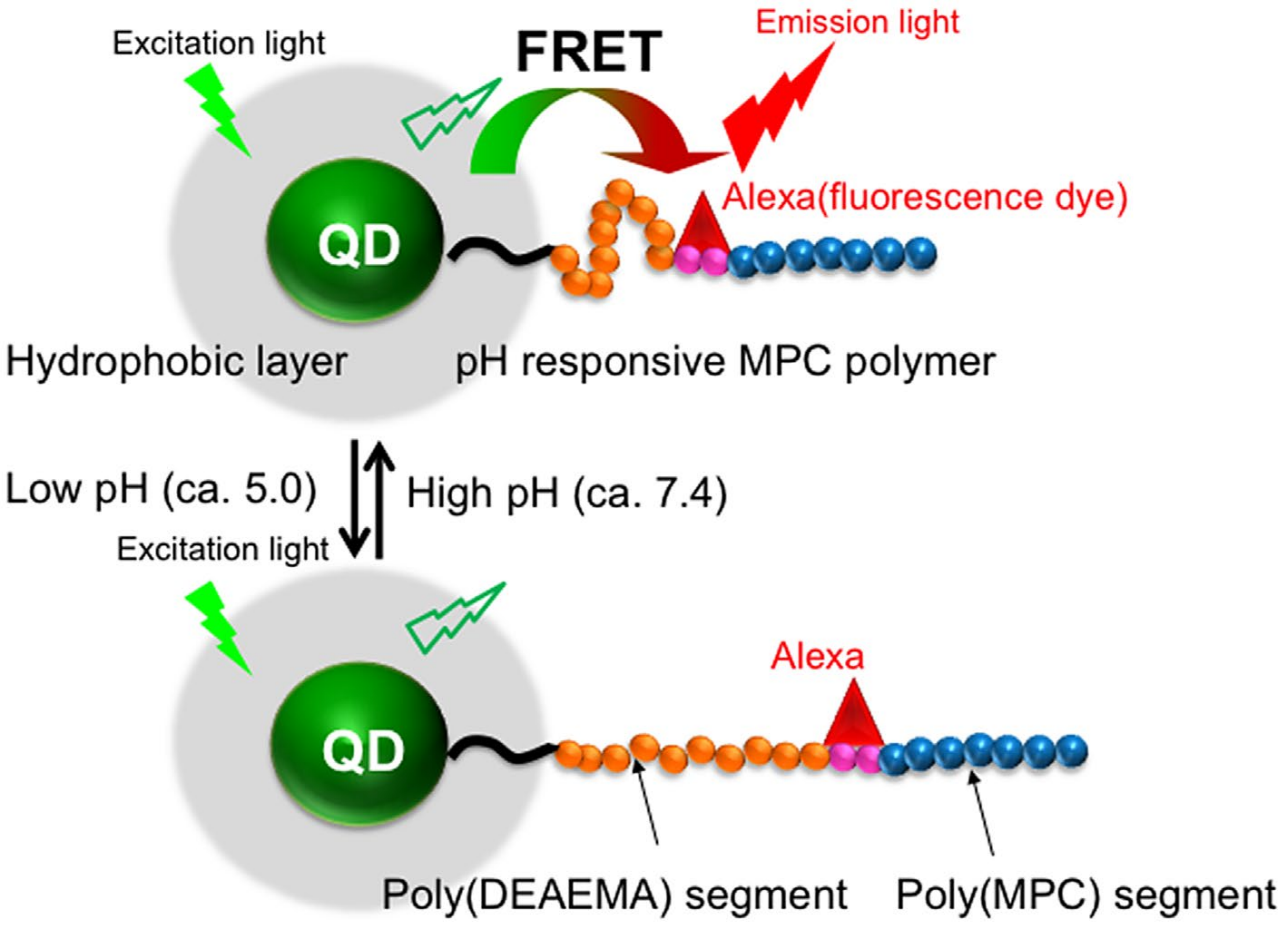
Schematic representation of QD modified with pH-responsive triblock-type MPC polymer and its FRET generation in response to pH change.

Liu et al. [74] reported the preparation of a fluorescent probe for evaluating pH conditions in cells, which showed a combination of pH response and changes in fluorescence spectra (Figure 3). The nanoparticles had a core-shell type structure with a QD core and a block-type water-soluble polymer composed of a poly(*N*,*N*-diethylaminoethyl methacrylate (DEAEMA)) segment, a poly(ω-(*p*-nitrophenyloxycarbonyl oligo(ethylene glycol)) methacrylate (MEONP)) segment, and a poly(MPC) segment as the shell. The poly(DEAEMA) segment could induce a stretch-shrink transformation with a change in the pH of the medium due to protonation of the DEAEMA units. An organic fluorescent dye, Alexa 594 cadaverine, was immobilized in the poly(MEONP) segment in the block-type polymer. The nanoparticles could disperse well in an aqueous medium and fluorescence resonance energy transfer (FRET) between the QD core and fluorescent dye was observed in the media at pH values of 7.4 and 5.0. This change in pH corresponded to an endocytosis process known as the proton sponge effect. The fluorescence spectrum was significantly altered between pH 7.4 and 5.0 because the distance between the QDs and fluorescent dye changed according to the pKa (7.1) of the pH-responsive poly(DEAEMA) segment. Thus, when the distance between the QDs and fluorescent dye was within several nanometers at pH 7.4, FRET was induced from the QDs (the donor) to the fluorescent dye (the acceptor). This produced an increase in the fluorescence intensity of the red fluorescent dye. Alternatively, when the distance increased at pH 5.0 due to protonation of the polymer DMAEMA units, independent fluorescence of the QDs and fluorescent dye were observed. To enhance the cellular uptake of the MPC polymer-covered QDs, one of the cell-penetrating peptides (CPPs),[95,96] octaarginine (R8), was immobilized at the terminal of the poly(MPC) segment. After addition to the cell culture medium followed by 35 min of incubation, the nanoparticles were internalized into the cells by endocytosis, and fluorescence from both the QDs and fluorescent dye was observed due to the formation of FRET. However, after 70 min of incubation, the fluorescence from the fluorescent dye disappeared in response to the decrease in the pH value. Henceforth, after 105 min of incubation, the fluorescence from the fluorescent dye was recovered owing to escape of the nanoparticles from the endosomes. These nanoparticles find a promising application to monitor the live transportation process of molecules into cells, along with the possibility to track their pathway inside the cells continuously.

## 4. Entrapment of QDs in polymeric nanoparticles covered with MPC polymers

**Figure 4.**
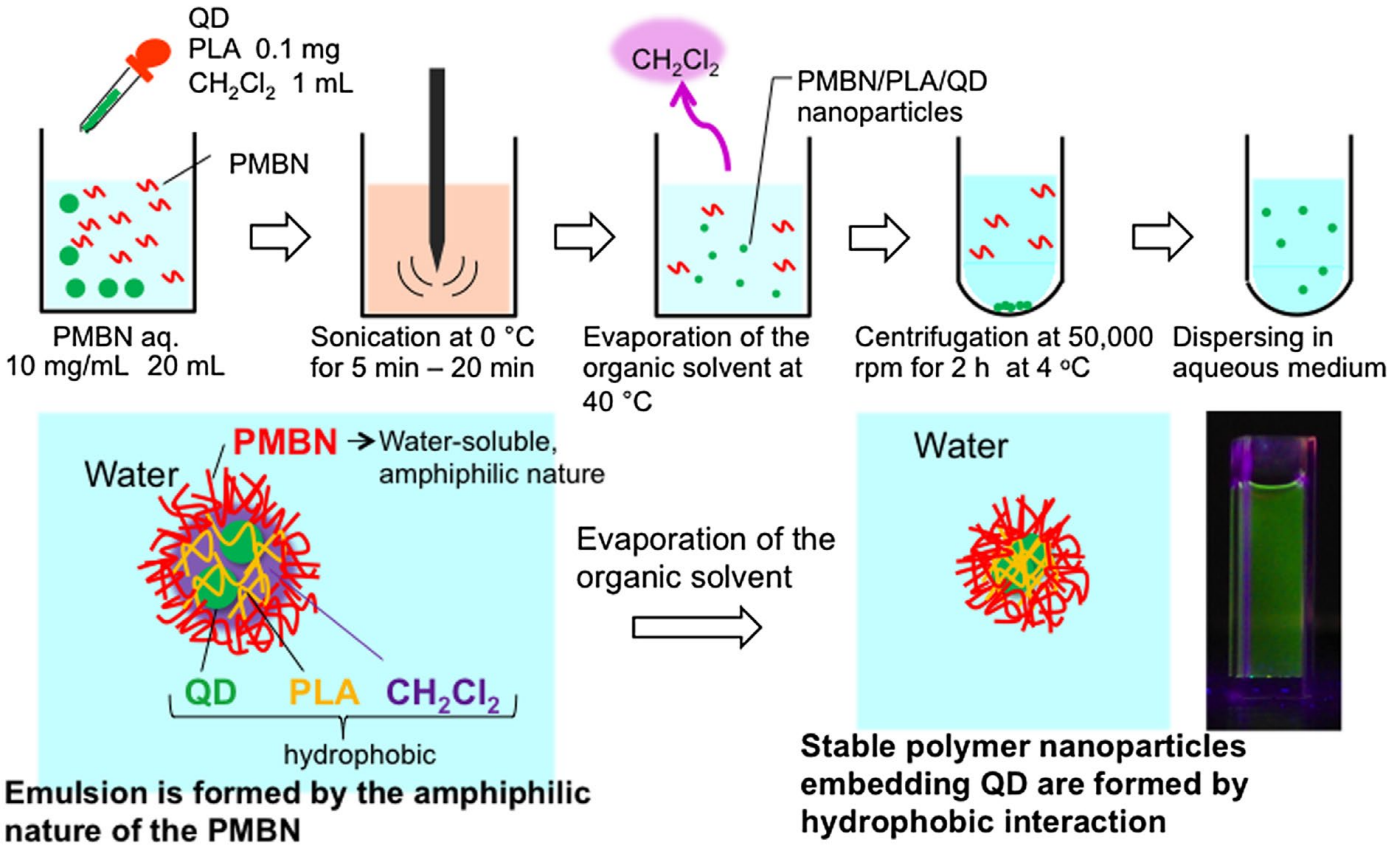
Schematic representation of the entrapment of QDs by PLA nanoparticles and coating with MPC polymers.

Previously, we prepared polymeric nanoparticles covered with MPC polymers in an aqueous solution using a simple solvent evaporation method, wherein amphiphilic MPC polymers were used as a suspension stabilizer. A hydrophobic polymer dissolved in an immiscible organic solvent was then added to the MPC polymer solution. Dichloromethane was used as the solvent due to its low boiling point and the hydrophobic polymers used were poly(L-lactic acid) (PLA) and polystyrene (PSt). The fabrication of polymer nanoparticles containing QDs was carried out using the solvent evaporation technique, which was simple and inexpensive (Figure 4).[76–78,97–100] Hydrophobic interactions among the materials play an important role in the mechanism by which the amphiphilic MPC polymers, PLA, and QDs form nanoparticles. The core of the nanoparticles composed of PLA and QDs, which are insoluble in water, was solubilized in dichloromethane. During the solvent evaporation process, evaporation of dichloromethane resulted in the precipitation of hydrophobic PLA chains with QDs at the interface of the aqueous medium. Additionally, at this interface, the MPC polymer chains formed entanglements with PLA chains, resulting in the formation of a stable MPC polymer layer on the surface of the PLA core.

**Figure 5.**
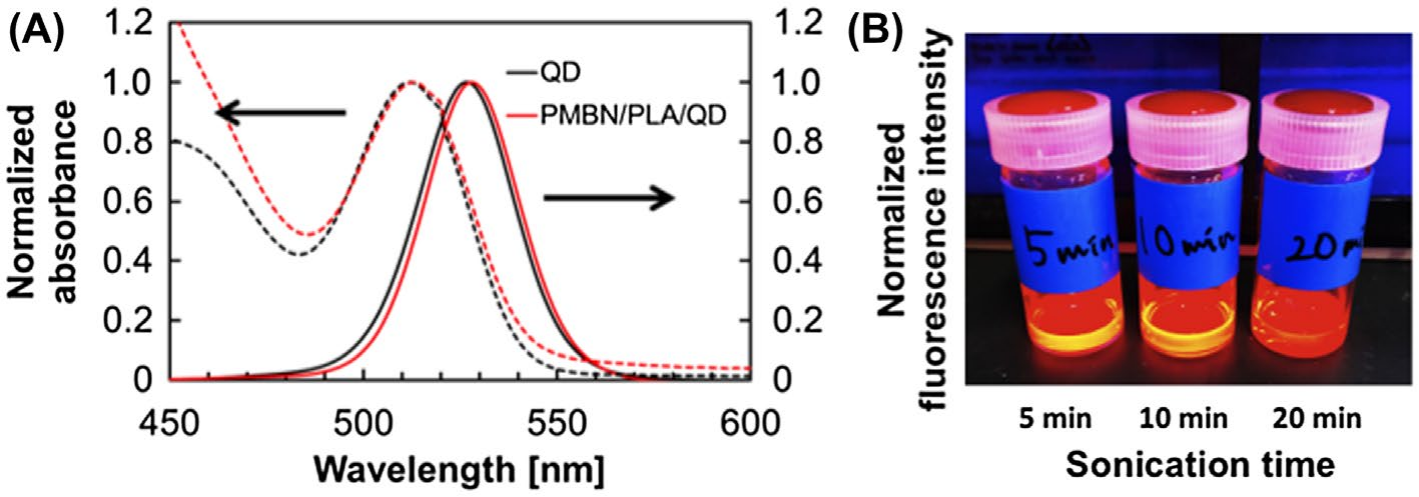
Fluorescence spectra of the QD and PMBN/PLA/QD nanoparticles (A), and fluorescence image of the PMBN/PLA/QD nanoparticles prepared with various sonication times (B).

**Figure 6.**
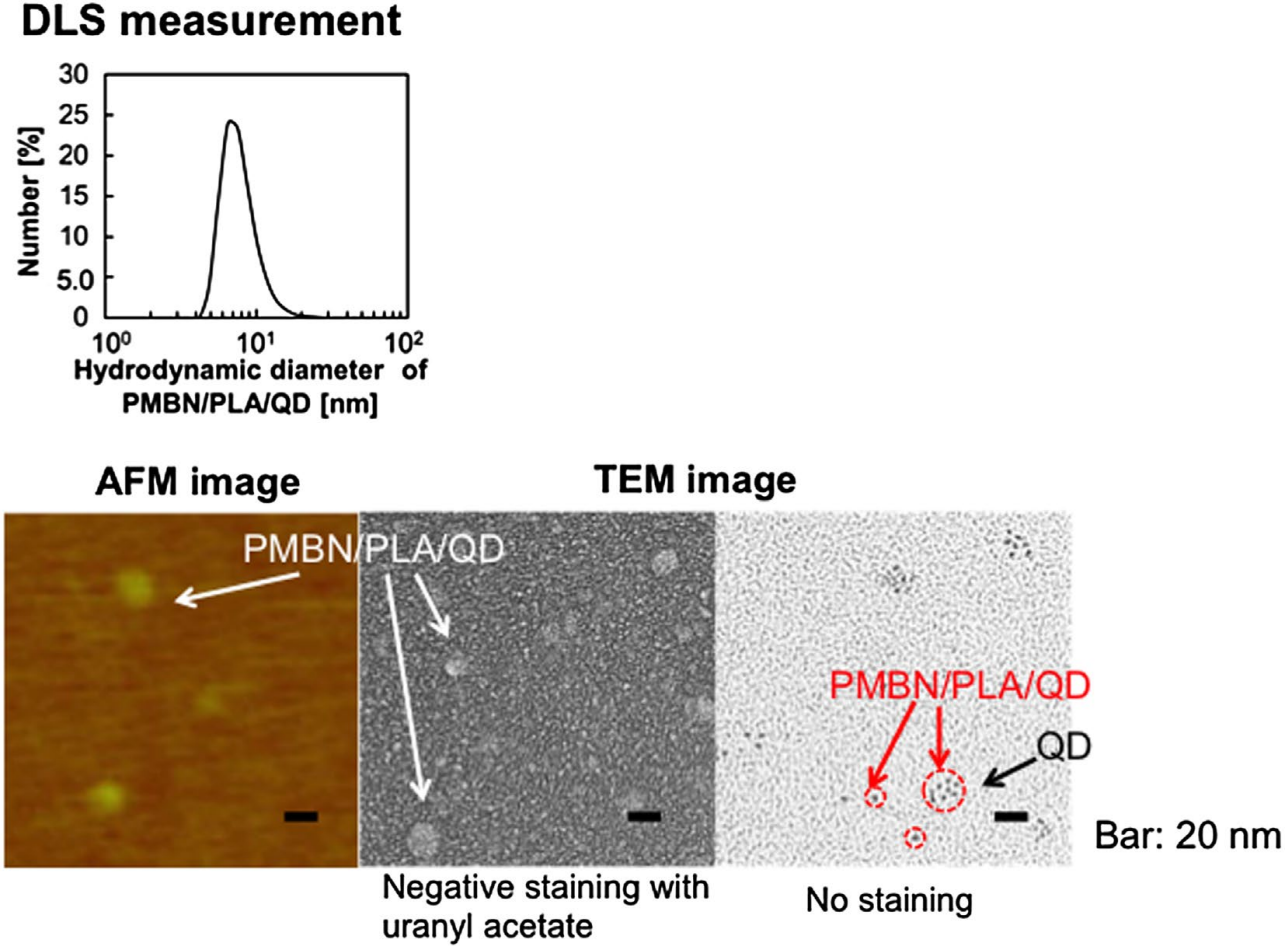
Morphological observation of PMBN/PLA/QD nanoparticles.

Instead of MPC polymer, the use of poly(MPC-*co*-*n*-butyl methacrylate (BMA)-*co*-NEONP) (PMBN) was analyzed.[97,101] Dissolution of PMBN in aqueous medium indicated an amphiphilic nature and, due to the presence of hydrophobic BMA units, tended to form polymer aggregates at a PMBN concentration of 0.10 g dl^–1^. Dichloroethylene droplets containing PLA and QDs were dispersed in the PMBN solution by sonication. During the preparation process, the sonication time was controlled to maintain the fluorescence intensity of the QDs. The PMBN/PLA/QD nanoparticles showed good dispersibility in aqueous medium, such as phosphate buffered saline and cell culture medium. The ultraviolet absorption and emission fluorescence spectra of QDs in the polymer nanoparticles were the same as those of commercially available QDs suspended in toluene (Figure 5). It was confirmed that the entrapment of QDs provided good long-term stability with no change in either solubility or fluorescence performance, even beyond six months of storage at 4°C. The PMBN/PLA/QD nanoparticles functioned well between pH 4.0 and 9.0. Photobleaching examination revealed that PMBN/PLA/QD nanoparticles were more resistant to continuous excitation photoirradiation compared with an organic fluorescent dye, fluorescein isothiocyanate. The entrapment of QDs using PLA and PMBN had no influence on the optical properties of the QDs. The hydrodynamic diameters of the PMBN/PLA/QD nanoparticles were around 10–20 nm, as measured by dynamic light scattering (DLS), and the size distribution was found to be narrow. Atomic force microscopy (AFM) showed that the morphology of PMBN/PLA/QD nanoparticles was spherical. According to AFM observations, the size measurements were consistent with the hydrodynamic diameter determined by DLS measurements. Moreover, transmission electron microscopy (TEM) revealed several groups of QDs appearing as dark spherical objects, a result of the electron-dense QDs being entrapped within a single polymer nanoparticle (Figure 6). X-ray photoelectron spectroscopy (XPS) analysis indicated that the PMBN/PLA/QD nanoparticles had specific XPS signals at 133 eV and 403 eV, which corresponded to the phosphorus atom in the phosphate group and the nitrogen atom in the ammonium group of the MPC unit, respectively. Thus, from these findings, the surface of PMBN/PLA/QD nanoparticles was covered with phosphorylcholine groups in the MPC polymer.

## 5. Transport of MPC polymer nanoparticles with biomolecules into cells

**Figure 7.**
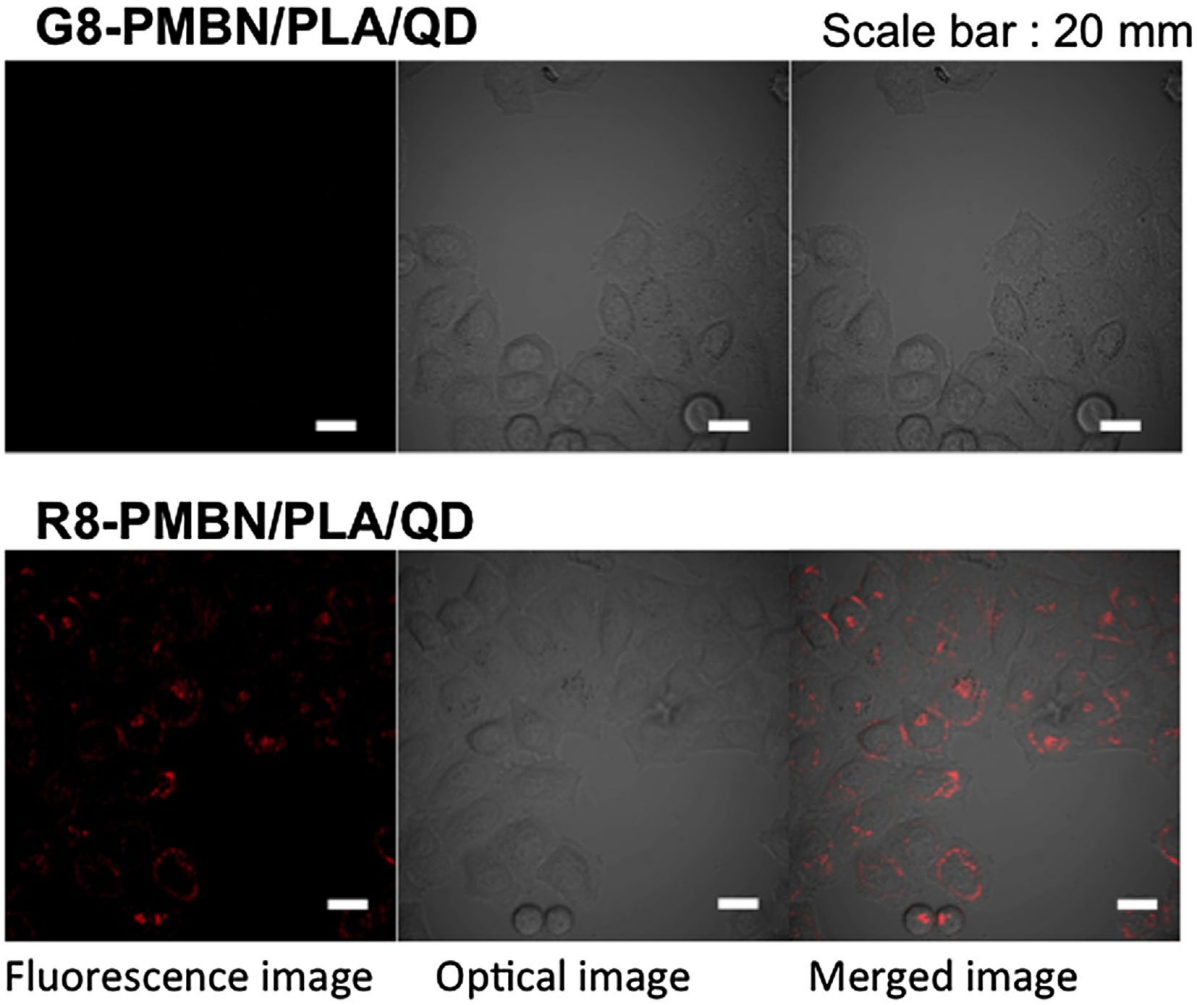
Uptake of the octapeptide-PMBN/PLA/QD nanoparticles by HeLa cells.

Immobilization of biomolecules on the PNBN/PLA/QD nanoparticles is important for successfully penetrating the cell membrane. R8, a well-known CPP,[95,96] and octaglycine (G8) were independently immobilized onto the surfaces of nanoparticles and applied to cell culture medium of cultured HeLa cells. As shown in Figure 7, G8-immobilized PMBN/PLA/QD nanoparticles (G8-PMBN/PLA/QD) were not internalized in the HeLa cells, even after incubation for 24 h. However, the R8-immobilized PMBN/PLA/QD nanoparticles (R8-PMBN/PLA/QD) were internalized effectively into the cells. The difference between these two nanoparticles indicated that the PMBN/PLA/QD nanoparticles without signal oligopeptides could provide the highest signal to noise ratio among all existing imaging probes, as there was no background fluorescence due to nonspecific uptake of imaging probes by the cells. This is due to the excellent cytocompatibility of the MPC polymers covering the nanoparticles.[36–42]

The internalization process of R8-PMBN/PLA/QD nanoparticles by the cells was examined. The R8-PMBN/PLA/QD nanoparticles adhered to the cell membrane within the first 5 min. Then, internalization of the nanoparticles into endosomes was initiated over the next 15–30 min. The amount of internalized R8-PMBN/PLA/QD nanoparticles increased from 1–3 h. All R8-PMBN/PLA/QD nanoparticles adhered to the cell membrane were internalized by the endosomes within 5 h. These results provided the kinetic information of the R8-mediated internalization of nanoparticles into the cell.

Cytotoxicity analysis indicated that PMBN/PLA/QD nanoparticles had no cytotoxicity three days post internalization into the HeLa cells. However, another possibility was induction of an inflammatory response. Further studies were required with respect to the inflammatory responses. This was analyzed by detecting the relative expression of TNF-α mRNA to GAPDH mRNA in RAW264.7 cells after incubation with R8-PMBN/PLA/QD nanoparticles or G8-PMBN/PLA/QD nanoparticles for a day. No significant difference was observed. This suggested that the MPC polymers on the surface of the PMBN/PLA/QD nanoparticles suppressed the inflammatory response in RAW264.7 cells during the CPP-mediated internalization into cells. These findings clearly showed that any undesirable interactions between PMBN/PLA/QD nanoparticles and the cells, such as non-specific uptake, cytotoxic effects, or induced inflammatory responses, were eliminated.

**Figure 8.**
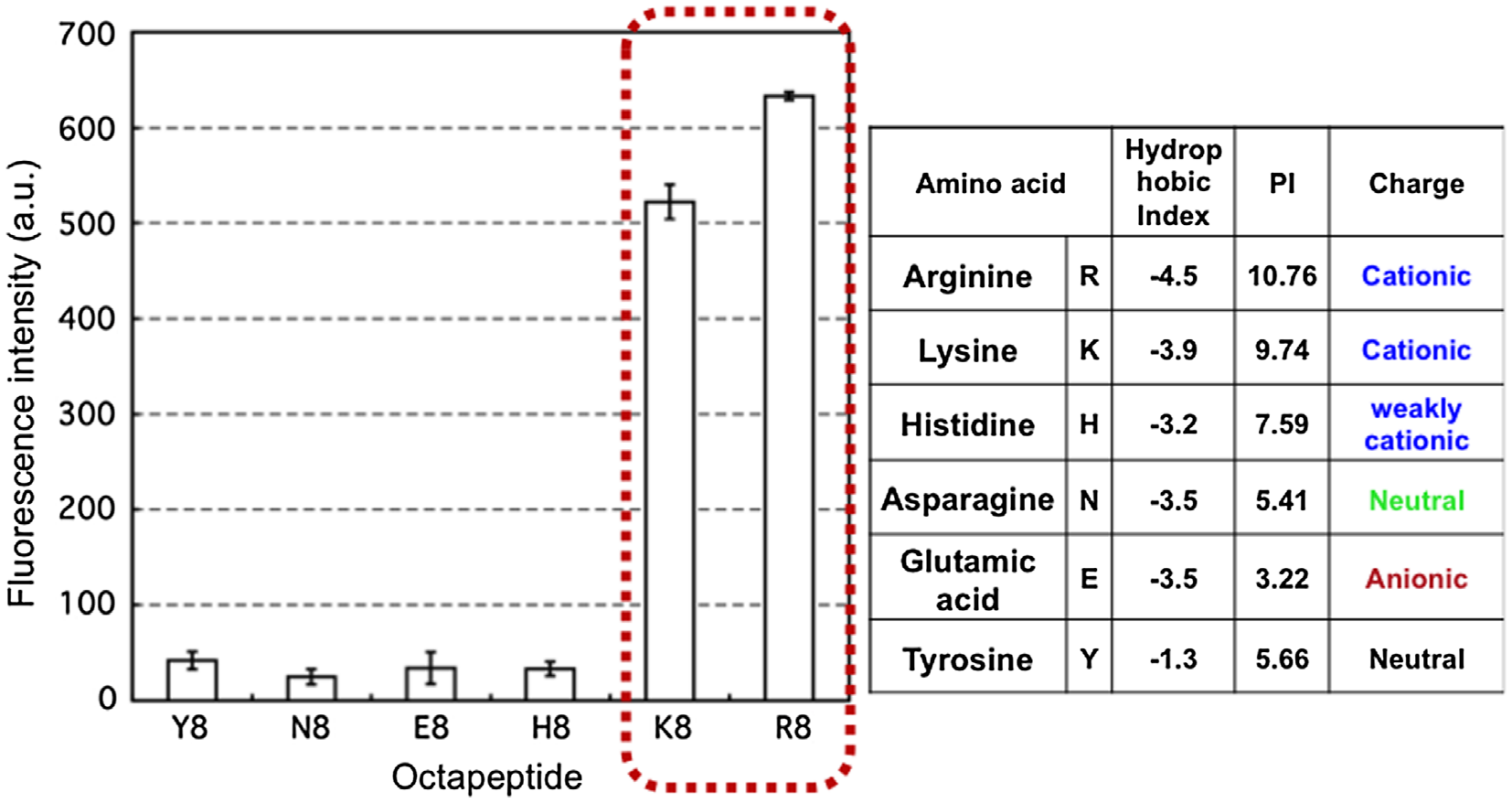
Effect of the chemical structure of octapeptide immobilized on PMBN/PLA/QD nanoparticles on cellular internalization. (Reproduced from [102] with copyright permission, Wiley-VCH)

Arginine-rich peptides are typically used as CPPs. The effects of other oligopeptides on permeation through the cell membrane are not well understood. We evaluated the role of various octapeptides immobilized on the PMBN/PLA/QD nanoparticles (Figure 8).[102] The selected octapeptide had simple sequences of only one kind of amino acid, hydrophobic and neutral tyrosine (Y8), hydrophilic and neutral asparagine (N8), anionic glutamic acid (E8), weakly cationic histidine (H8), cationic lysine (K8), and mostly hydrophilic and cationic arginine (R8). Of the octapeptides tested, only K8- and R8-immobilized PMBN/PLA/QD nanoparticles could be internalized into HeLa cells. These findings were supported by the report of another research group arriving at the same result that R8 and K8 octapeptides could function as CPPs. These results suggest that molecules functioning as CPPs should possess hydrophilic and electron donating properties.

**Figure 9.**
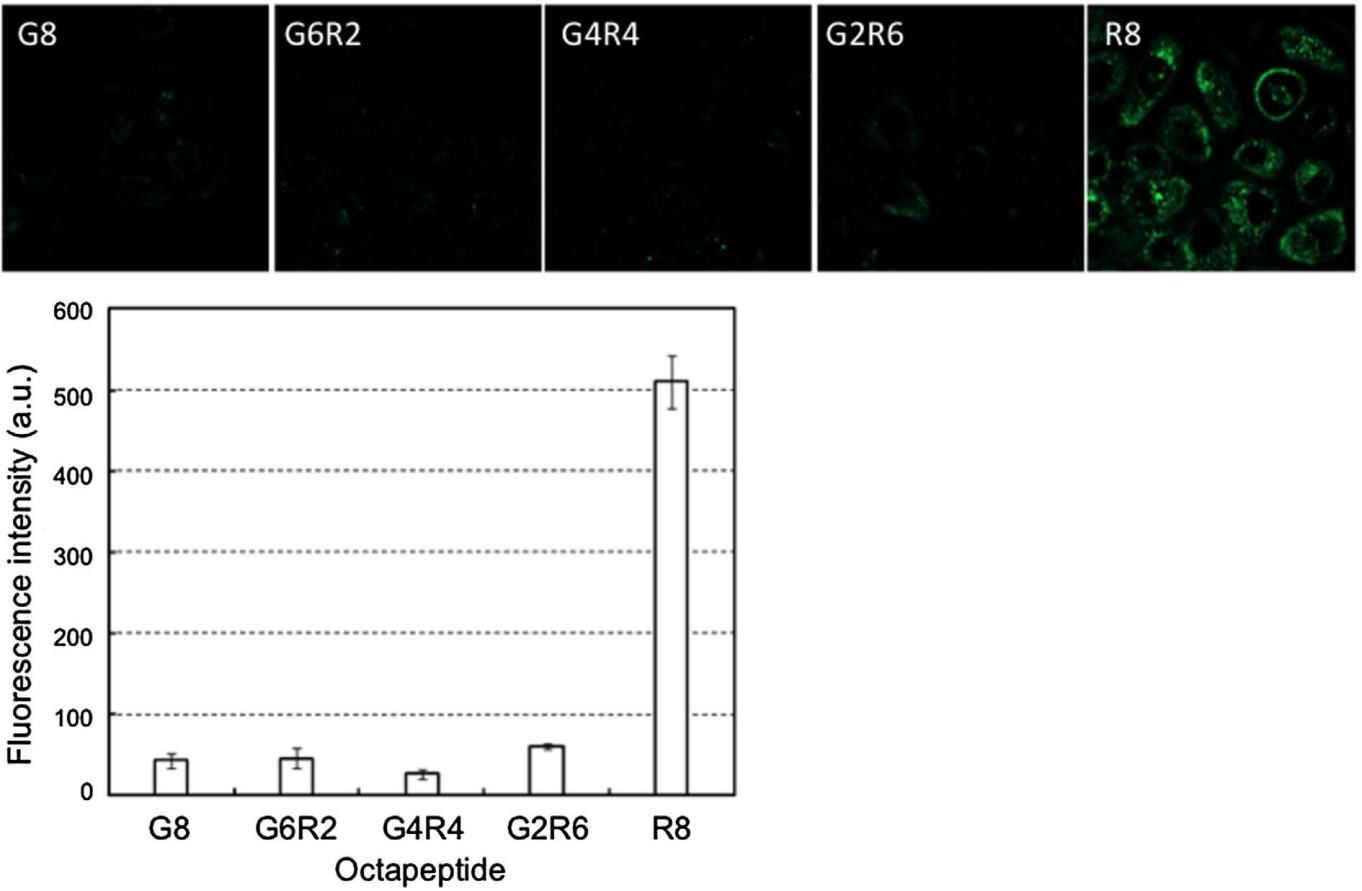
Effect of the sequence of octapeptide immobilized on PMBN/PLA/QD nanoparticles on cellular internalization. Internalization of nanoparticles was evaluated by fluorescence microscopy (upper panel), and the fluorescence intensity was determined for each nanoparticles (lower panel). (Reproduced from [101].)

**Figure 10.**
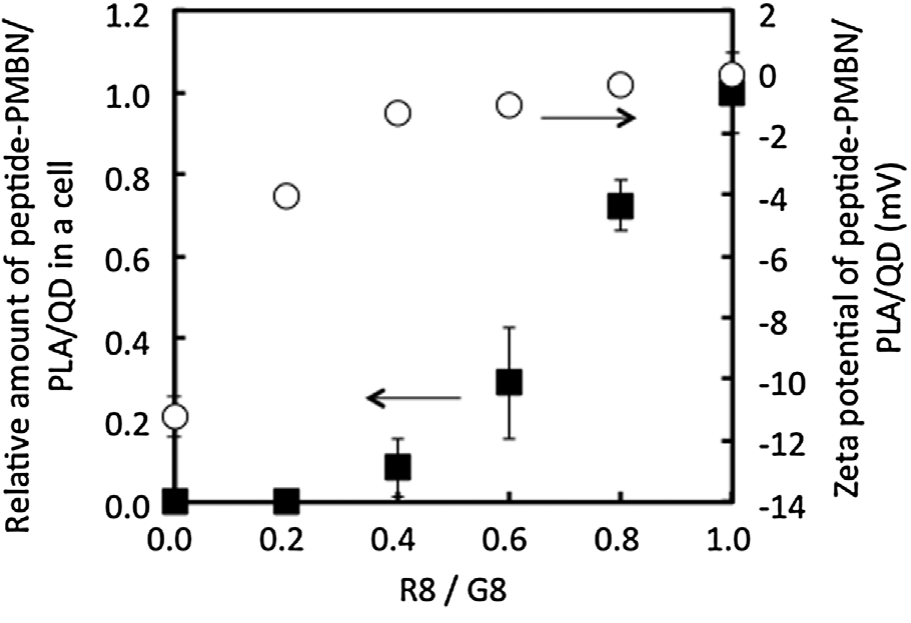
Effect of composition of R8 and G8 immobilized on PMBN/PLA/QD nanoparticles on cellular internalization.

To understand the effect of the sequence of octapeptides composed of inert and active amino acid residues for the process of internalization into cells, the following series of octapeptides were prepared using glycine (G) and arginine (R) and analyzed: G8, GGGGGGRR (G6R2), GGGGRRRR (G4R4), GGRRRRRR (G2R6), and R8. Figure 9 represents images of cells examined by fluorescence microscopy. Addition of the respective octapeptide-coated nanoparticles to cell culture medium initiated cellular contact between the nanoparticles and the cultured cells. The results indicated that only the R8-immobilized nanoparticles could be internalized into cells. Next, the effect of the surface density of R8-mediated internalization of nanoparticles into cells was evaluated. To achieve this, the ratio of the concentration of R8 to G8 in the feed solution during immobilization was altered to control the surface density of R8. The results of the internalization of the nanoparticles are shown in Figure 10. Although the surface ζ-potential of G8-PNBN/PLA/QD nanoparticles was slightly negative (–11 mV), it increased correspondingly with an increase in the R8 fraction until a ratio of 0.4. However, up to the R8/G8 ratio of 0.4, the surface ζ-potential was constant around 0 mV. On the other hand, the amount of nanoparticles internalized into the cells increased around the R8/G8 ratio of 0.4 and a significant increase was observed up to the R8/G8 ratio of 0.6. These results were interesting, as they suggested that surface charge was not an essential factor for internalization of nanoparticles into the cells. This opens up the scope for further research and consideration to analyze possible molecular interactions between the oligopeptide unit and the cell membrane surface. PMBN/PLA/QD nanoparticles not only inhibit nonspecific cellular uptake by mammalian cells, but also provide insight about specific interactions between biomolecules and cells when bioactive molecules like R8 are immobilized on them. From these results, it is concluded that R8-PMBN/PLA/QD nanoparticles are a promising probe for high sensitivity and non-inflictive cell and tissue imaging technology.

## 6. Behavior of R8-immobilized MPC polymer nanoparticles *in vivo*

**Figure 11.**
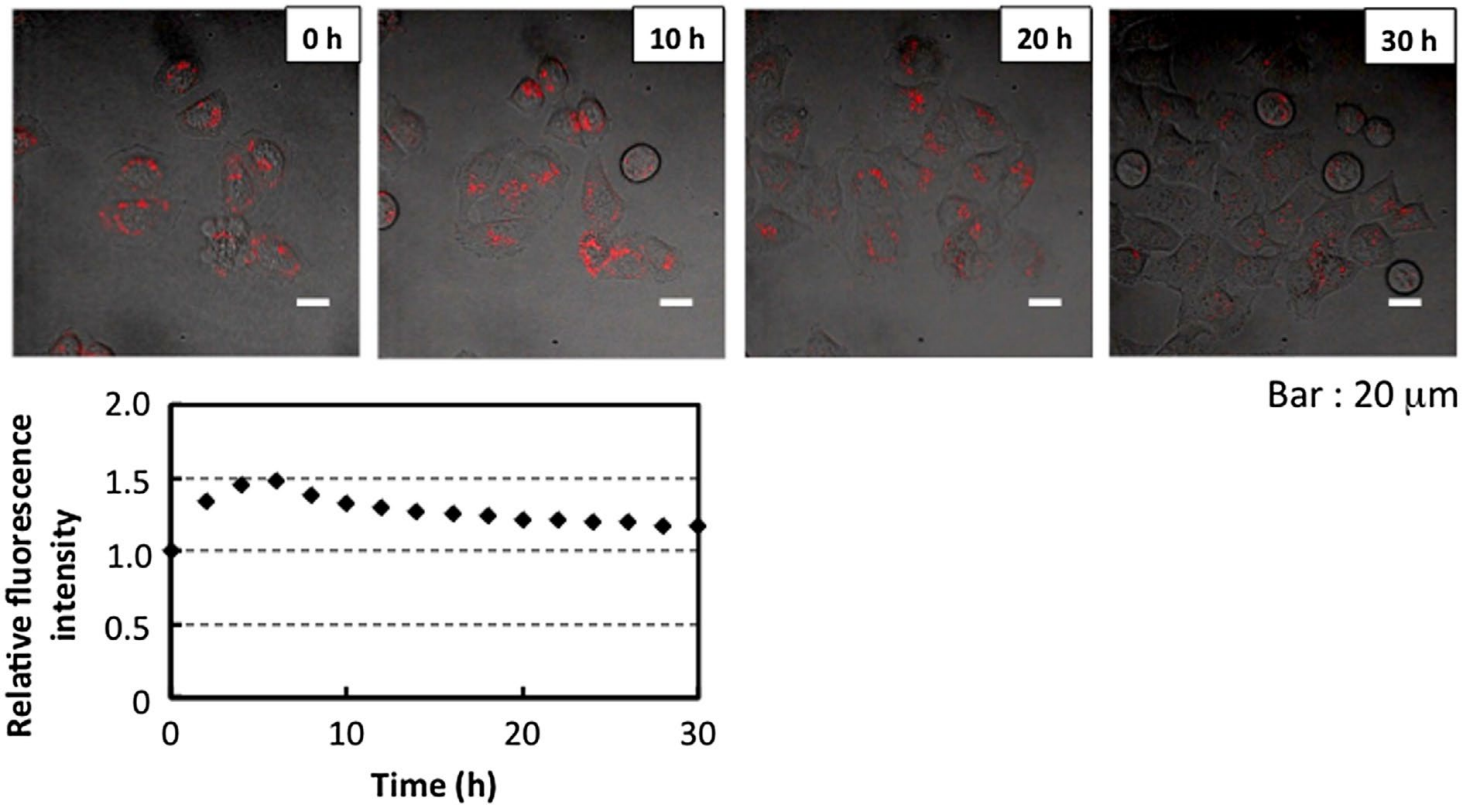
Distribution of R8-PMBN/PLA/QD nanoparticles in the cells during cell proliferation (upper panel) and relative fluorescence intensity of total microscope area (lower panel). (Reproduced from [102].)

Observation of the dynamics of nanoparticles in cells is important for understanding the typical endocytosis pathways of internalization. The R8-PMBN/PLA/QD nanoparticles associated with HeLa cells significantly.[97,102] No change in cell proliferation was observed compared with proliferation of the original cells for more than 30 h of cell culture. The distribution of nanoparticles was observed by fluorescence microscopy during cell proliferation as shown in Figure 11. During the proliferation process, the R8-PMBN/PLA/QD nanoparticles in the cells were distributed to two daughter cells by cell proliferation, and the amount of R8-PMBN/PLA/QD nanoparticles in each cell decreased. Correspondingly, the fluorescence intensity of the R8-PMBN/PLA/QD nanoparticles in all the cells did not change for about 30 h.[102] This was the first report indicating long-term retention of nanoparticles inside cells.

## 7. Future perspectives

Useful applications of nanobioengineering would not be achievable without the suitable design of nanostructures for materials and devices. Continued progress in biomedical nanotechnology requires additional systematic approaches for the chemical and physical design of nanostructures. Moreover, the interface between the nanostructure and biological environments becomes critical as researchers start to construct multifunctional nanostructures.

The design of the chemical structure of MPC polymers was strongly inspired by natural phospholipids at the cell membrane surface. This feedback from biological systems represents a rational strategy for obtaining new polymeric biomaterials. The development of MPC polymers did not have limitations in the induction of cell and tissue responses in biological systems. Thus, many kinds of MPC polymers have been designed and synthesized with flexibility for use in several applications. The PMBN described in this review is a useful polymer for constructing artificial cell membrane structures. By combining specific biomolecules on the PMBN surface, it can be used as a delivery system for biomolecules, as well as for the development of diagnostic and bioimaging probes. MPC units that are concentrated on the surface of nanodevices provide a high shielding effect against protein adsorption. The effect of MPC units does not interrupt the reactivity of immobilized biomolecules on the surfaces. The conjugation of biomolecules on the MPC polymer surface with a desirable arrangement is essential in achieving an excellent biointerface. The artificial cell membrane structure constructed by the MPC polymer and biomolecules can be applied for the preparation of in-cell nanodevices with the ability to completely control the interactions between nanodevices and cells. These facts underline the importance of biointerface technology in understanding the fundamentals of biomolecular behavior in cellular environments.

## Disclosure statement

No potential conflict of interest was reported by the authors.

## References

[CIT0001] Kim JW, Cho J, Cho J (2016). Synthesis of monodisperse bi-compartmentalized amphiphilic janus microparticles for tailored assembly at the oil–water interface Angew. Chem Int Ed.

[CIT0002] Abe H, Liu J, Ariga K (2016). Catalytic nanoarchitectonics for environmentally compatible energy generation. Mater Today.

[CIT0003] Shin T-H, Choi Y, Kim S (2015). Recent advances in magnetic nanoparticle-based multi-modal imaging. Chem Soc Rev.

[CIT0004] Wang H, Liu S, Zhang Y-L (2015). Controllable assembly of silver nanoparticles induced by femtosecond laser direct writing. Sci Technol Adv Mater.

[CIT0005] Sheng Y, De Liao LD, Thakor NV (2014). Nanoparticles for molecular imaging. J Biomed Nanotechnol.

[CIT0006] Tang Y, Cheng W (2015). Key parameters governing metallic nanoparticle electrocatalysis. Nanoscale.

[CIT0007] Wu W, Wu Z, Yu T (2015). Recent progress on magnetic iron oxide nanoparticles: synthesis, surface functional strategies and biomedical applications. Sci Technol Adv Mater.

[CIT0008] Kim S-J, Xu W, Ahmad MD (2015). Synthesis of nanoparticle CT contrast agents: *in vitro* and *in vivo* studies. Sci Technol Adv Mater.

[CIT0009] Zeth K, Hoiczyk E, Okuda M (2016). Ferroxidase-mediated iron oxide biomineralization: novel pathways to multifunctional nanoparticles. Trends Biochem. Sci.

[CIT0010] Wang Z, Yu J, Gui R (2016). Carbon nanomaterials-based electrochemical aptasensors. Biosens Bioelectron.

[CIT0011] Kandasamy G, Maity D (2015). Recent advances in superparamagnetic iron oxide nanoparticles (SPIONs) for *in vitro* and *in vivo* cancer nanotheranostics. Int J Pharm.

[CIT0012] Samiei M, Farjami A, Dizaj SM (2016). Nanoparticles for antimicrobial purposes in Endodontics: A systematic review of *in vitro* studies. Mater Sci Eng C Mater Biol.

[CIT0013] Passos ML, Pinto PC, Santos JL (2015). Nanoparticle-based assays in automated flow systems: a review. Anal Chim Acta.

[CIT0014] Mou X, Ali Z, Li S (2015). Applications of magnetic nanoparticles in targeted drug delivery system. J Nanosci Nanotechnol.

[CIT0015] Mekaru H, Lu J, Tamanoi F (2015). Development of mesoporous silica-based nanoparticles with controlled release capability for cancer therapy. Adv Drug Deliv Rev.

[CIT0016] Loos C, Syrovets T, Musyanovych A (2014). Functionalized polystyrene nanoparticles as a platform for studying bio-nano interactions. Beilstein J Nanotechnol.

[CIT0017] Pissuwan D, Niidome T (2015). Polyelectrolyte-coated gold nanorods and their biomedical applications. Nanoscale.

[CIT0018] Bernsen MR, Guenoun J, van Tiel ST (2015). Nanoparticles and clinically applicable cell tracking. Br J Radiol.

[CIT0019] Soenen SJ, Parak WJ, Rejman J (2015). (Intra)Cellular stability of inorganic nanoparticles: effects on cytotoxicity, particle functionality, and biomedical applications. Chem Rev.

[CIT0020] Doh KO, Yeo Y (2012). Application of polysaccharides for surface modification of nanomedicines. Ther Deliv.

[CIT0021] Yang H-M, Park C-W, Ahn T (2013). A direct surface modification of iron oxide nanoparticles with various poly(amino acid)s for use as magnetic resonance probes. J Colloid Interface Sci.

[CIT0022] Huynh R, Chaubet F, Jozefonvicz J (2001). Anticoagulant properties of dextranmethylcarboxylate benzylamide sulfate (DMCBSu); a new generation of bioactive functionalized dextran. Carbohydrate Res.

[CIT0023] Lemarchand C, Gref R, Couvreur P (2004). Polycaccharide-decorated nanoparticles. Eur J Pharm Biopharm.

[CIT0024] Rabanel J-M, Hildgen P, Banquy X (2014). Assessment of PEG on polymeric particles surface, a key step in drug carrier translation. J Controlled Release.

[CIT0025] Suk J-S, Xu Q, Kim N (2016). PEGylation as a strategy for improving nanoparticle-based drug and gene delivery. Adv Drug Delivery Rev.

[CIT0026] Shimizu T, Mima Y, Hashimoto Y (2015). Anti-PEG IgM and complement system are required for the association of second doses of PEGylated liposomes with splenic marginal zone B cells. Immunobiology.

[CIT0027] Mima Y, Hashimoto Y, Shimizu T (2015). Anti-PEG IgM is a major contributor to the accelerated blood clearance of polyethylene glycol-conjugated protein. Mol Pharm.

[CIT0028] Wang C, Cheng X, Su Y Accelerated blood clearance phenomenon upon cross-administration of PEGylated nanocarriers in beagle dogs. Int J Nanomedicine.

[CIT0029] Puri A, Blumenthal R (2011). Polymeric lipid assemblies as novel theranostic tools. Acc Chem Res.

[CIT0030] Monge S, Canniccioni B, Graillot A (2011). Phosphorus-containing polymers: a great opportunity for the biomedical field. Biomacromolecules.

[CIT0031] Ruiz L, Hilborn JG, Léonard D (1998). Synthesis, structure and surface dynamics of phosphorylcholine functional biomimicking polymers. Biomaterials.

[CIT0032] Nagase Y, Nakajima S, Oku M (2008). Synthesis and properties of segmented poly(urethane-urea)s containing phosphorylcholine moiety in the side-chain. Polym J.

[CIT0033] Ishihara K, Ueda T, Nakabayashi N (1990). Preparation of phospholipid polymers and properties as hydrogel membranes. Polym J.

[CIT0034] Ishihara K, Fukazawa K, Monge S, David G (2014). 2-Methacryloyloxyethyl phosphorylcholine polymer. Phosphorus-based polymers: from synthesis to applications.

[CIT0035] Ueda T, Oshida H, Kurita K (1992). Preparation of 2-methacryloyloxyethyl phosphorylcholine copolymers with alkyl methacrylates and their blood compatibility. Polym J.

[CIT0036] Ishihara K, Aragaki R, Ueda T (1990). Reduced thrombogenicity of polymers having phospholipid polar groups. J Biomed Mater Res.

[CIT0037] Ishihara K, Ziats NP, Tierney BP (1991). Protein adsorption from human plasma is reduced on phospholipid polymers. J Biomed Mater Res.

[CIT0038] Ishihara K, Nomura H, Mihara T (1998). Why do phospholipid polymer reduce protein adsorption ?. J Biomed Mater Res.

[CIT0039] Moro T, Takatori Y, Ishihara K (2004). Surface grafting of artificial joints with a biocompatible polymer for preventing periprosthetic osteolysis. Nat Mater.

[CIT0040] Ishihara K (2000). Bioinspired phospholipid polymer biomaterials for making high performance artificial organs. Sci Technol Adv Mater.

[CIT0041] Iwasaki Y, Ishihara K (2005). Phosphorylcholine-containing polymers for biomedical applications. Ann Bioanal Chem.

[CIT0042] Lewis AL, Lloyd AW, Santin M, Phillips G (2012). Biomedical applications of biomimetic polymers: the phosphorylcholine-containing polymers in biomimetic, bioresponsive, and bioactive materials. An Introduction to Integrating Materials with Tissues.

[CIT0043] Iwasaki Y, Ishihara K (2012). Cell membrane-inspired phospholipid polymers for developing medical devices with excellent biointerfaces. Sci Technol Adv Mater.

[CIT0044] Ishihara K (2015). Highly lubricated polymer interfaces for advanced artificial hip joints through biomimetic design. Polym J.

[CIT0045] Kihara S, Yamazaki K, Litwak KN (2003). In vivo evaluation of a mpc polymer coated continuous flow left ventricular assist system. Artif Organs.

[CIT0046] Lewis AL, Vick TA, Collias AC (2001). Phosphorylcholine-based polymer coatings for stent drug delivery. J Mater Sci Mater Med.

[CIT0047] Zhang Z, Cao X, Zhao X (2006). Controlled delivery of antisense oligodeoxynucleotide from cationically modified phosphorylcholine polymer films. Biomacromolecules.

[CIT0048] Zhong Q, Yan J, Qian X (2014). Atomic layer deposition enhanced grafting of phosphorylcholine on stainless steel for intravascular stents. Colloids Surf B Biointerfaces.

[CIT0049] Shimada T, Ueda M, Jinno H (2009). Development of targeted therapy with paclitaxel incorporated into EGF-conjugated nanoparticles. Anticancer Res.

[CIT0050] Miyata R, Ueda M, Jinno H (2009). Selective targeting by preS1 domain of hepatitis B surface antigen conjugated with phosphorylcholine-based amphiphilic block copolymer micelles as a biocompatible, drug delivery carrier for treatment of human hepatocellular carcinoma with paclitaxel. Int J Cancer.

[CIT0051] McRae Page SM, Henchey E, Chen X (2014). Efficacy of polyMPC–DOX prodrugs in 4t1 tumor-bearing mice. Mol Pharmaceutics.

[CIT0052] Kano T, Kakinuma C, Wada S (2011). Enhancement of drug solubility and absorption by copolymers of 2-Methacryloyloxyethyl phosphorylcholine and n-butyl methacrylate. Drug Metab Pharmacokinet.

[CIT0053] Onoue S, Kojo Y, Suzuki H (2013). Development of novel solid dispersion of tranilast using amphiphilic block copolymer for improved oral bioavailability. Int J Pharm.

[CIT0054] Onoue S, Suzuki H, Kojo Y (2014). Self-micellizing solid dispersion of cyclosporine A with improved dissolution and oral bioavailability. Eur J Pharm Sci.

[CIT0055] Ukawa M, Akita H, Masuda T (2010). 2-Methacryloyloxyethyl phosphorylcholine polymer (MPC)-coating improves the transfection activity of GALA-modified lipid nanoparticles by assisting the cellular uptake and intracellular dissociation of plasmid DNA in primary hepatocytes. Biomaterials.

[CIT0056] Wen Y, Zhang Z, Li J (2014). Highly efficient multifunctional supermolecular gene carrier system self-assembled from redox-sensitive and zwitterionic polymer blocks. Adv Funct Mater.

[CIT0057] Lam JK, Armes SP, Lewis AL (2009). Folate conjugated phosphorylcholine-based polycations for specific targeting in nucleic acids delivery. J Drug Target.

[CIT0058] Qiang G, Lee I (2011). Kinetics of swelling-shrinking rearrangement of a self-aggregated nanohydrogel at solid/liquid interfaces. J Exp Nanosci.

[CIT0059] Xu S, Ye Z, Wu P (2015). Biomimetic controlling of CaCO_3_ and BaCO_3_ superstructures by zwitterionic polymer. ACS Sustainable Chem Eng.

[CIT0060] Jin S, Zhou N, Xu D (2013). Synthesis and characterization of poly(2-methacryloyloxyethyl phosphorylcholine) onto graphene oxide. Polym Adv Technol.

[CIT0061] Dai F, Zhang M, Hu B (2015). Immunomagnetic nanoparticles based on a hydrophilic polymer coating for sensitive detection of Salmonella in raw milk by polymerase chain reaction. RSC Advence.

[CIT0062] Peacock AK, Cauët SI, Taylor A (2012). Poly[2-(methacryloyloxy)ethylphosphorylcholine]-coated iron oxide nanoparticles: synthesis, colloidal stability and evaluation for stem cell labelling. Chem Commun (Camb).

[CIT0063] Sun XY, Yu SS, Wan JQ (2013). Facile graft of poly(2-methacryloyloxyethyl phosphorylcholine) onto Fe(3) O(4) nanoparticles by ATRP: synthesis, properties, and biocompatibility. J Biomed Mater Res A.

[CIT0064] Li X, Zhang B, Tian L (2015). Improvement of recognition specificity of surface protein-imprinted magnetic microspheres by reducing nonspecific adsorption of competitors using 2-methacryloyloxyethyl phosphorylcholine. Snes Actu B: Chemical.

[CIT0065] Yokoyama R, Suzuki S, Shirai K (2006). Preparation and properties of biocompatible polymer-grafted silica nanoparticle. Eur Polym J.

[CIT0066] Müllner M, Cui J, Noi KF (2014). Surface-initiated polymerization within mesoporous silica spheres for the modular design of charge-neutral polymer particles. Langmuir.

[CIT0067] Shao Z, Yang Y, Lee H (2012). Synthesis and suspension rheology of titania nanoparticles grafted with zwitterionic polymer brushes. J Colloid Interface Sci.

[CIT0068] Fuchs AV, Walter C, Landfester K (2012). Biomimetic silver-containing colloids of poly(2-methacryloyloxyethyl phosphorylcholine) and their film-Formation properties. Langmuir.

[CIT0069] Fuchs AV, Ritz S, Putz S Bioinspired phosphorylcholine containing polymer films with silver nanoparticles combining antifouling and antibacterial properties. Biomater Sci.

[CIT0070] Kitayama Y, Takeuchi T (2014). Localized surface plasmon resonance nanosensing of C-reactive protein with poly(2-methacryloyloxyethyl phosphorylcholine)-grafted gold nanoparticles prepared by surface-initiated atom transfer radical polymerization. Anal Chem.

[CIT0071] Chen X, Lawrence J, Parelkar S (2013). Novel zwitterionic copolymers with dihydrolipoic acid: synthesis and preparation of nonfouling nanorods. Macromolecules.

[CIT0072] Bortolotto T, Facchinetto ST, Trindade SG (2015). Polymer-coated palladium nanoparticle catalysts for Suzuki coupling reactions. J Colloid Interface Sci.

[CIT0073] Matsuno R, Goto Y, Konno T (2009). Controllable nanostructured surface modification on quantum dot for biomedical application in aqueous medium. J Nanosci Nanotechnol.

[CIT0074] Liu Y, Inoue Y, Ishihara K (2015). Surface functionalization of quantum dots with fine-structured pH-sensitive phospholipid polymer chains. Colloids Surf B: Biointerfaces.

[CIT0075] Xu FM, Xu JP, Lv L-P (2011). Bowl- and porous sphere-shaped supramolecular assemblies and their application as templates for confirmed assembly of gold nanoparticles. Soft Matter.

[CIT0076] Goto Y, Matsuno R, Konno T (2008). Polymer nanoparticles covered with phosphorylcholine groups and immobilized with antibody for high-affinity separation of proteins. Biomacromolecules.

[CIT0077] Kim HI, Ishihara K (2014). Phospholipid polymer can reduce cytocompatibility of poly(lactic acid) nanoparticles in a high-content screening assay. Biomater Biomed Engineer.

[CIT0078] Chen W, Inoue Y, Ishihara K (2015). Preparation of photoreactive phospholipid polymer nanoparticles to immobilize and release protein by photoirradiation. Colloids Surf B: Biointerfaces.

[CIT0079] Osawa K, Imae T, Ujihara M (2013). Preparation of amphiphilic diblock copolymers with pendant hydrophilic phosphorylcholine and hydrophobic dendron groups and their self-association behavior in water. J Polym Sci Polym. Chem.

[CIT0080] Han H, Zhang S, Wang Y (2016). Biomimetic drug nanocarriers prepared by miniemulsion polymerization for near-infrared imaging and photothermal therapy. Polymer.

[CIT0081] Ishihara K, Iwasaki Y, Nakabayashi N (1999). Polymeric lipid nanosphere constituted of poly(2-methacryloyloxyethyl phosphorylcholine-*co*-*n*-butyl methacrylate) Polym. J.

[CIT0082] Konno T, Ishihara K (2003). Enhanced solubility of paclitaxel using water-soluble and biocompatible 2-methacryloyloxyethyl phosphorylcholine polymers. J Biomed Mater Res.

[CIT0083] Lobb EJ, Ma I, Billingham NC (2001). Facile synthesis of well-defined, biocompatible phosphorylcholine-based methacrylate copolymers via atom transfer radical polymerization at 20 °C. J Am Chem Soc.

[CIT0084] Yusa S-I, Fukuda K, Yamamoto T (2005). Synthesis of well-defined amphiphilic block copolymers having phospholipid polymer sequences as a novel biocompatible polymer micelle reagent. Biomacromolecules.

[CIT0085] Shen L (2011). Biocompatible polymer/quantum dots hybrid materials: current status and future developments. J Funct Biomater.

[CIT0086] Wegner KD, Hildebrandt N (2015). Quantum dots: bright and versatile *in vitro* and *in vivo* fluorescence imaging biosensors. Chem Soc Rev.

[CIT0087] Wang Y, Hu R, Lin G (2013). Functionalized quantum dots for biosensing and bioimaging and concerns on toxicity. ACS Appl Mater Interfaces.

[CIT0088] Tripathi SK, Kaur G, Khurana RK (2015). Quantum dots and their potential role in cancer theranostics. Crit Rev Ther Drug Carrier Syst.

[CIT0089] Petryayeva E, Algar WR, Medintz IL (2013). Quantum dots in bioanalysis: a review of applications across various platforms for fluorescence spectroscopy and imaging. Appl Spectrosc.

[CIT0090] Hu K, Wang H, Tang G (2015). In vivo cancer dual-targeting and dual-modality imaging with functionalized quantum dots. J Nucl Med.

[CIT0091] Liu H, Tang W, Li C (2015). CdSe/ZnS quantum dots-labeled mesenchymal stem cells for targeted fluorescence imaging of pancreas tissues and therapy of type 1 diabetic rats. Nanoscale Res Lett.

[CIT0092] Breus VV, Pietuch A, Tarantola M (2015). The effect of surface charge on nonspecific uptake and cytotoxicity of CdSe/ZnS core/shell quantum dots. Beilstein J Nanotechnol.

[CIT0093] Painuly D, Bhatt A, Krishnan VK (2014). Physicochemical and *in vitro* biocompatibility evaluation of water-soluble CdSe/ZnS core/shell. J Biomater Appl.

[CIT0094] Jańczewski D, Tomczak N, Han M-Y (2011). Synthesis of functionalized amphiphilic polymers for coating quantum dots. Nat Protocols.

[CIT0095] Reissmann S (2014). Cell penetration: scope and limitations by the application of cell-penetrating peptides. J Pept Sci.

[CIT0096] Brock R (2014). The uptake of arginine-rich cell-penetrating peptides: putting the puzzle together. Bioconjug Chem.

[CIT0097] Goto Y, Matsuno R, Konno T (2008). Artificial cell membrane-covered nanoparticles embedding quantum dots as stable and highly sensitive fluorescence bioimaging probes. Biomacromolecules.

[CIT0098] Watanabe J, Ishihara K (2007). Instantaneous determination via bimolecular recognition: usefulness of FRET in phosphorylcholine group enriched nanoparticles. Bioconjug Chem.

[CIT0099] Konno T, Watanabe J, Ishihara K (2004). Conjugation of enzymes on polymer nanoparticles covered with phosphorylcholine groups. Biomacromolecules.

[CIT0100] Ito T, Watanabe J, Takai M (2006). Dual mode bioreactions on polymer nanoparticles covered with phosphorylcholine group. Colloids Surf B Biointerfaces.

[CIT0101] Ishihara K, Tsukamoto Y, Goto Y (2013). Enhanced and specific internalization of polymeric nanoparticles to cells. IFMBE Proceedings.

[CIT0102] Ishihara K, Ruiz-Molina D, Novio F, Roscini C (2015). Novel bioinspired phospholipid polymer biomaterials for nanobioengineering. Bio- and bioinspired nanomaterials.

